# Supplementary material for: Multicomponent molecular memory
Source: Nat Commun. 2020 Feb 4;11:691. doi: 10.1038/s41467-020-14455-1 (PMC7000828; doi:10.1038/s41467-020-14455-1)
Supplement: Supplementary file 1 — Description of Additional Supplementary Files [file 41467_2020_14455_MOESM1_ESM.pdf]

Title: Supplementary Data 1

Description: A complete table of the Ugi library.
