# Supplementary material for: Multicomponent molecular memory
Source: Nat Commun. 2020 Feb 4;11:691. doi: 10.1038/s41467-020-14455-1 (PMC7000828; doi:10.1038/s41467-020-14455-1)
Supplement: Supplementary file 3 — Supplementary Information [file 41467_2020_14455_MOESM3_ESM.pdf]

Supplementary Information for

Multicomponent Molecular Memory

Christopher E. Arcadia, Eamonn Kennedy, Joseph Geiser, Amanda Dombroski, Kady Oakley, Shui-Ling Chen, Leonard Sprague, Mustafa Ozmen, Jason Sello, Peter M. Weber, Sherief Reda, Christopher Rose, Eunsuk Kim, Brenda M. Rubenstein, and Jacob K. Rosenstein
Brown University, Providence, RI, USA

December 23, 2019

Contents

Supplementary Methods **3**
1 Ugi Product Library . . . 3
1.1 Library Synthesis . . . 3
1.2 Library Subset Selection . . . 7
1.3 Other Synthesized Ugi Products . . . 7
2 Data Storage . . . 9
2.1 Writing Data . . . 9
2.2 Data Written . . . 10
2.3 Long-Term Storage . . . 11
2.4 Comparison to Related Work . . . 13
3 Mass Spectrometry . . . 14
3.1 Spectrum Acquisition . . . 14
3.2 Mass Calibration . . . 14
3.3 Spot Alignment . . . 16
3.4 Repeated Reads . . . 17
3.5 Signal-to-Noise Estimation . . . 18
3.6 Adduct Ions . . . 19
4 Parametric Sweeps . . . 20
4.1 Spot Size . . . 20
4.2 Acquisition Duration . . . 21
4.3 Laser Power . . . 22
4.4 Ugi Product Concentration . . . 24

Supplementary References **25**

Supplementary Methods

1. Ugi Product Library

1.1. Library Synthesis

To make a library, the reagents, shown in Figure 1, are manually prepared as 500 mM solutions in dimethyl sulfoxide (DMSO) and 60 μL of each is pipetted into a 384-well plate. To level the fluid menisci, we spin the plate in a centrifuge for five minutes at 2,500 rpm. The reagent plate is then placed into an Echo 550 acoustic liquid dispenser, surveyed to check volume levels, and finally used to prepare the reaction wells according to a pre-generated list of transfers. Maps indicating where each reagent was placed on an example library plate are shown in Figure 2e. The reagents are added by class, in the following order: amines, aldehydes, carboxylic acids, and lastly isocyanides. The resulting reaction wells, filled with 200 nL of each reagent solution (800 nL in total), are left to react. After an incubation period of about 24-48 hours, solvent (DMSO) is added to bring the final product volumes to 4 μL. Images of the reagent, product, and MALDI plates for the Ugi library used in this work are provided in Figure 2. Additionally, the molecular structures of all 1,500 expected products are shown in Figure 3. We generally use these library elements for data storage without further purification. Over the course of this work, we have run over 10,000 Ugi reactions, up to 1,500 at a time.

Class	PubChem ID	Monoisotopic Mass [Da]	Name	Structure
Aldehyde	16275	112.089	cyclohexane-carboxaldehyde	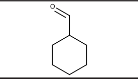
	78070	140.12	3-cyclohexyl-propanal	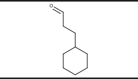
	8063	86.073	valeradlehyde	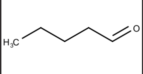
	11552	86.073	isovaleraldehyde	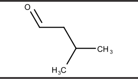
	70106	98.073	cyclopentane-carboxaldehyde	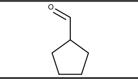
Amine	7504	107.073	benzylamine	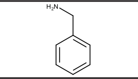
	66035	121.089	4-methyl-benzylamine	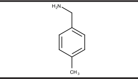
	75452	137.084	p-methoxy-benzylamine	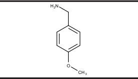
	66036	141.035	4-chloro-benzylamine	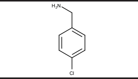
	2735655	163.136	4-tert-butyl-benzylamine	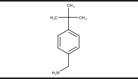
Isocyanide	79129	109.089	cyclohexyl isocyanide	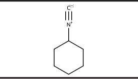
	533707	113.048	ethyl isocyanoacetate	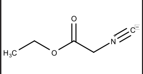
	82558	117.058	benzyl isocyanide	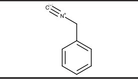
	16217475	153.058	2-naphthyl isocyanide	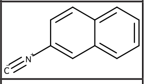
	547815	99.032	methyl isocyanoacetate	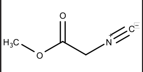

Class	PubChem ID	Monoisotopic Mass [Da]	Name	Structure
Carboxylic Acid	78288	175.084	Boc-glycine	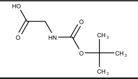
	85083	215.116	Boc-proline	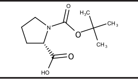
	7010608	231.147	Boc-N-methyl-L-valine	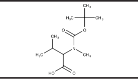
	82035	232.106	Boc-L-asparagine	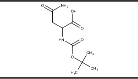
	2761526	245.163	Boc-L-beta-homoleucine	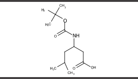
	89857	249.103	Boc-L-methionine	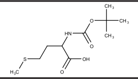
	2761810	260.137	Boc-L-beta-homo-glutamine	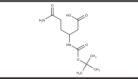
	16211045	263.119	Boc-L-betahomo-methionine	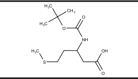
	77857	265.131	Boc-L-phenylalanine	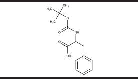
	1581890	274.153	Boc-N-alpha-N-epsilon-formyl-L-lysine	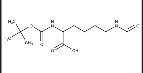
	7010602	279.147	Boc-N-methyl-L-phenylalanine	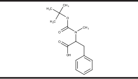
	2762280	295.142	Boc-O-methyl-L-tyrosine	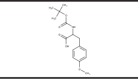

Supplementary Figure 1: A table of the reagents used to make the Ugi library. Unique combinations of the 5 aldehydes, 5 amines, 5 isocyanides, and 12 carboxylic acids yield 1,500 different Ugi products.

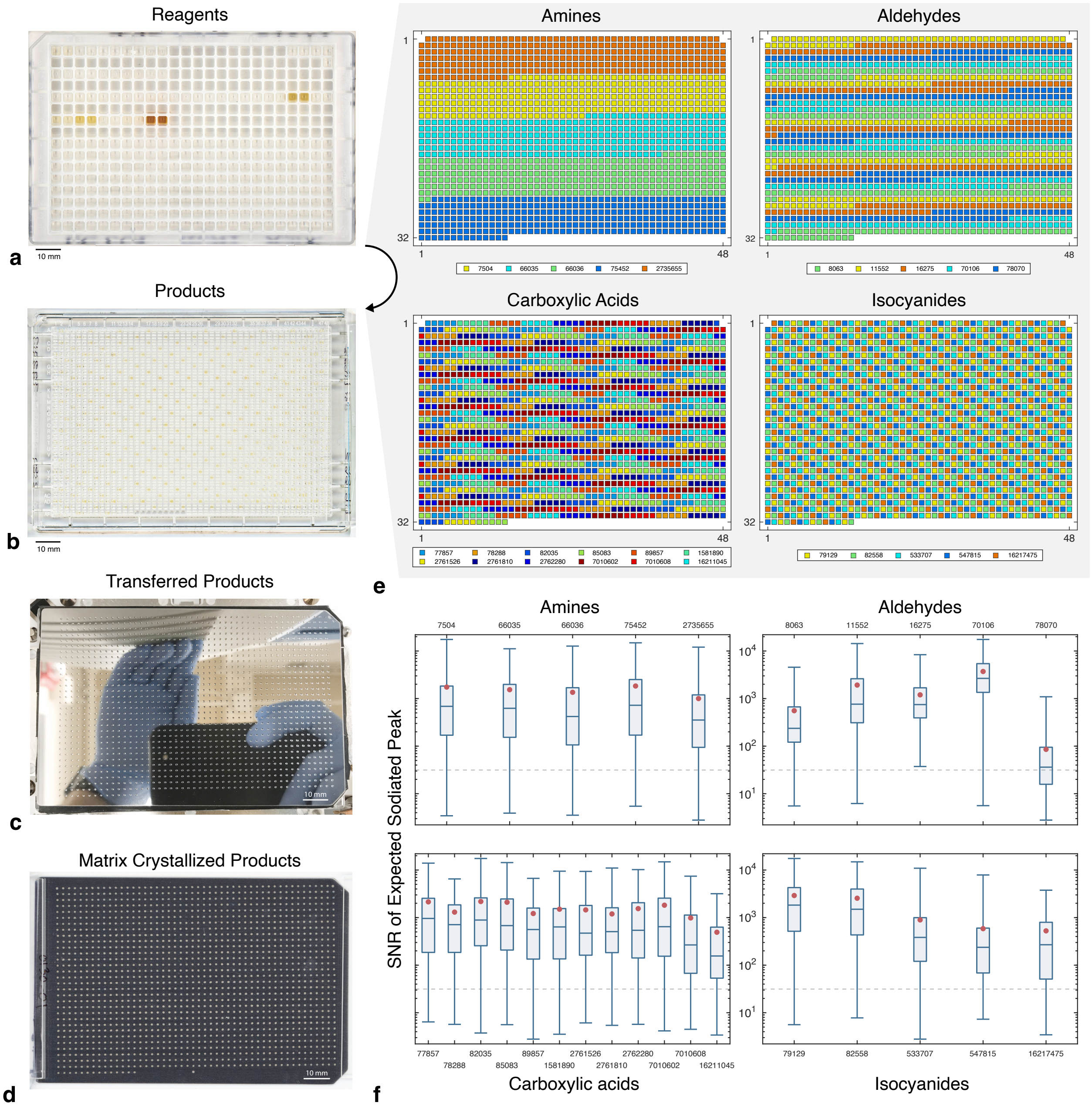

Supplementary Figure 2: Ugi product synthesis. **a** The initial 384-well source plate containing all reagents and solvent needed to make the library plate. **b** The final 1536-well library plate with all 1,500 unique reaction solutions. **c** Library contents after being sampled onto a stainless steel MALDI plate. **d** Library contents after drying on the MALDI plate. **e** Maps indicating where each reagent was placed on the library plate. **f** Reagent performance boxplots, which show product peak SNR statistics for the products made from each reagent. Here whiskers mark the maximums and minimums, box limits are the 1st and 3rd quartiles, box center lines are the medians, and circles indicate the means. Reagents are specified by their PubChem IDs.

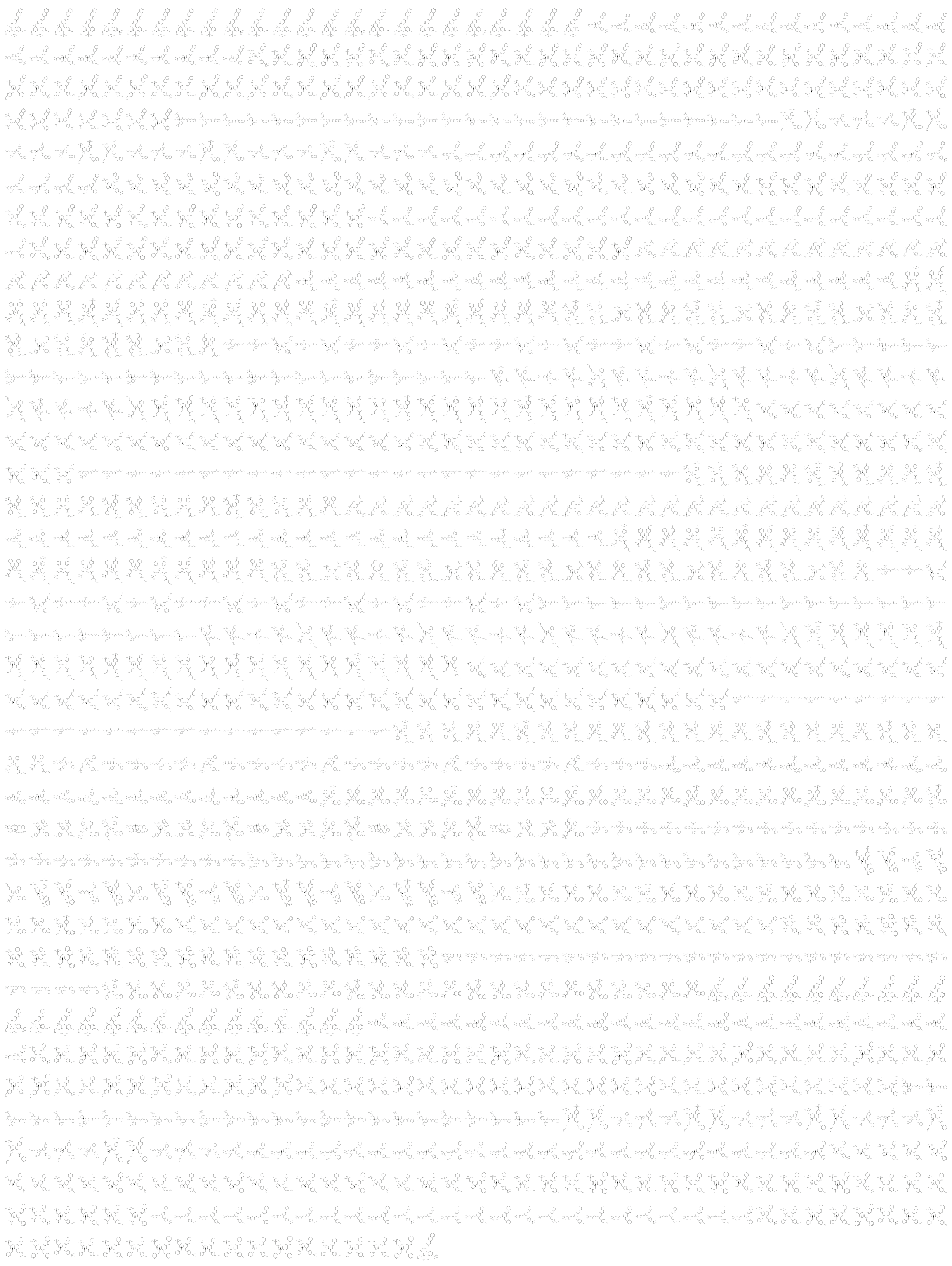

Supplementary Figure 3: The molecular structures of all 1,500 expected compounds in the Ugi library.

1.2. Library Subset Selection

The data storage examples described have all used a subset of the compound library, due in large part to the limited write speed. To select a subset, we begin with the list of library products having confirmed M+Na peaks. In the example below, 90% (1346/1500) were detected. We then sort these compounds by sodiated peak intensity. Starting from the strongest peak, we include each successive compound when its monoisotopic mass has a distance of at least 0.008 Da from any previously selected compounds. This is repeated until the desired number of compounds are obtained. An overview of the selection process is given in Figure 4. For the 32 products used in Figure 4, the average mass separation was 3.970 Da and the average SNR was approximately 10,000. Although we have not focused on such scenarios in this work, we note that by using multi-peak detection we can distinguish library elements with overlapping monoisotopic masses.

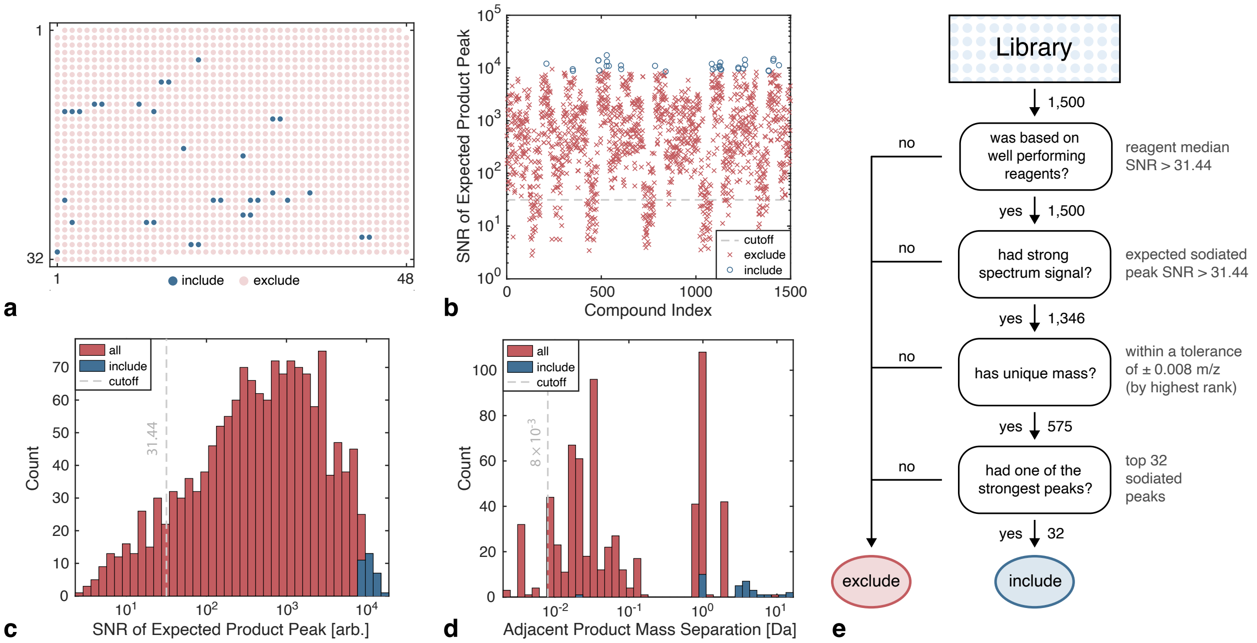

Supplementary Figure 4: Library subset selection. The example shown here is for the 32 compound Anubis storage demonstration. **a** Plate map of the selected library subset. **b** The mass intensities (SNR, sodiated) of the selected products, alongside that of the rest of the library. **c** A histogram of all library product signals (SNR, sodiated) and the optimal threshold (31.44) used to assess product presence. **d** A histogram of the differences between adjacent compound masses which illustrates the degree of mass diversity among library elements. Some products had non-unique masses and are not displayed on this log-scale chart. **e** A flow chart of the library refinement process.

1.3. Other Synthesized Ugi Products

For preliminary testing and calibration, several Ugi products were synthesized on a larger scale and purified using a silica column. A table of these products and the reagents used to make them is shown in Figure 5. One of these purified products (F13) is included in

all samples throughout this work as a reference mass to correct for sample-to-sample m/z drift. The others were used to test experimental conditions, such as laser power, sample and matrix concentration, and sample wear-out.

Name	Amine	Aldehyde	Carboxlic Acid	Isocyanide	Molecular Formula	Monoisotopic Mass [Da]	Recovered Mass [mg]	Recovered Yield [%]	Structure
C4	paramethoxy-benzylamine (75452)	2-nitro-benzaldehyde (11101)	Boc-L-phenylalanine (77857)	cyclohexyl isocyanide (79129)	$C_{36}H_{44}N_4O_7$	644.321	205.5	64	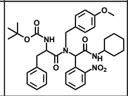
D6	paramethoxy-benzylamine (75452)	2-nitro-benzaldehyde (11101)	Boc-O-benzyl-L-tyrosine (98763)	benzyl isocyanide (82558)	$C_{44}H_{46}N_4O_8$	758.332	155.2	41	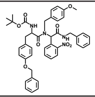
D9	paramethoxy-benzylamine (75452)	2-nitro-benzaldehyde (11101)	Boc-L-methionine (89857)	benzyl isocyanide (82558)	$C_{33}H_{40}N_4O_7S$	636.262	204	64	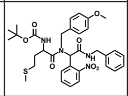
H6	paramethoxy-benzylamine (75452)	iso-butyraldehyde (6561)	Boc-O-benzyl-L-tyrosine (98763)	cyclohexyl isocyanide (79129)	$C_{40}H_{53}N_3O_6$	671.393	191.8	57	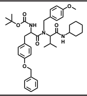
F13*	benzylamine (7504)	iso-butyraldehyde (6561)	Boc-glycine (78288)	tert-butyl isocyanide (23577)	$C_{23}H_{37}N_3O_4$	419.278	60.5	12	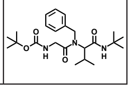

Supplementary Figure 5: A table of several manually prepared and purified Ugi products and their respective reagents. These products were synthesized using concentrated (4 M) reagents dissolved in dichloromethane (DCM). Reaction solutions were purified using a silica column, dried under vacuum, and reconstituted in dimethyl sulfoxide (DMSO) before use. The reagents and their PubChem IDs are provided for each product. *F13 is included in all samples and used as a mass lock for m/z offset calibration.

2. Data Storage

2.1. Writing Data

To write a dataset, compounds from a library plate are used to create mixtures corresponding to the data to be stored, by dispensing droplets with an acoustic liquid handler. The time required to write a dataset depends on the several factors, including the size of the data, the encoding scheme, the rate of droplet dispensing, and the time that the liquid handler takes to move between library wells. Figure 6a illustrates the write process. The liquid transfer lists are sorted by library source well and then destination well, in order to save time. Figure 6b and 6c plot the instantaneous and cumulative transfers from a library well to many locations on the Anubis data plate. The transfer rate was about 4 compounds/sec, which corresponded to a write speed of 18 bits/sec for this particular dataset.

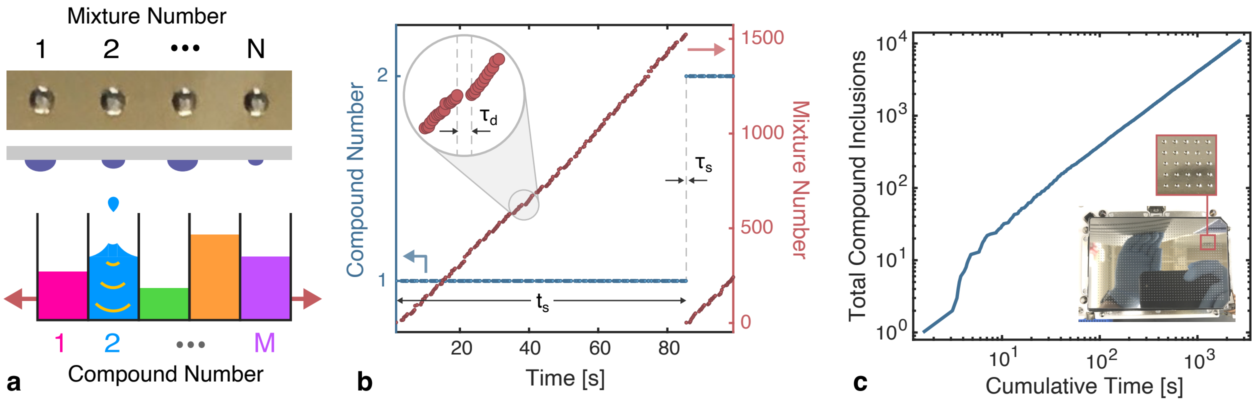

Supplementary Figure 6: Data mixture writing. **a** An illustration of the write process. Compounds from a library plate are used to generate mixtures corresponding to the digital data, by dispensing droplets with an acoustic liquid handler. **b** The library compound and data mixture wells addressed for each transfer, within a hundred second time-frame. **c** The cumulative number of compound transfers performed over time. Inset: An image of the produced data plate.

2.2. Data Written

Filename	Ibex	Anubis	Dimna	Angels	Violin
Original Data	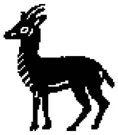	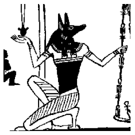	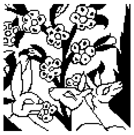	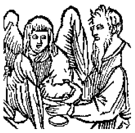	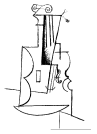
Size [bits]	6,142	48,841	12,100	24,336	879,625
Shape [px^2]	83×74	221×221	110×110	156×156	1135×775
Library Size	5	32	256	512	575
Mixture Complexity	0 - 5 (mean: 3.27)	0 - 32 (mean: 7.35)	16	32	0 - 575 (mean: 43.45)
Mixtures Used	1229	1527	1513	1521	1530
Write Time [hr]	0.328	1.299	3.306	6.131	7.860
Read Time [hr]	3.117	3.902	2.538	2.540	3.911
Times Written	×2	×1	×1	×1	×2
Times Read	×9	×1	×1	×5	×8
Mapping	direct	direct	sparse (2^8 entries)	sparse (2^{16} entries)	direct
Detection Method	single peak (sodiated)	single peak (sodiated)	single peak (sodiated)	multi-peak (logistic regression)	multi-peak (random forest regression)
Accuracy [%]	99.870	97.895	99.017 (raw: 84.637)	99.889 (raw: 95.236)	97.570
Recovered Data	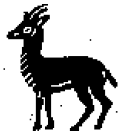	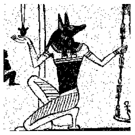	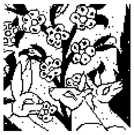	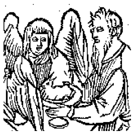	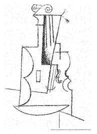

Supplementary Figure 7: A detailed summary of the data written into Ugi products mixtures. Experimental parameters are provided along with the original and recovered digital images. The images were adapted from artwork from The Metropolitan Museum of Art (Anubis [1], Dimna [2], Angels [3]) and [4] the © Estate of Pablo Picasso/Artists Rights Society (ARS), New York (Violin [4]).

2.3. Long-Term Storage

During the normal course of our experiments, we measure data plates within two weeks of writing them. However, the datasets are stable for much longer than that. For example, one of our earliest data plates (Ibex, Figure 8), has been read multiple times over the course of its year-long existence with no significant change in readout quality. Because the solvent has been evaporated and the molecules are trapped within matrix crystals, the stored data should remain intact as long as the samples are kept under reasonable environmental conditions. Some parameters which may affect the lifetime of stored data include:

- **Temperature:** The melting points of the matrix and encoding compounds represent a limit to the temperature a data plate can be exposed to before crystal integrity becomes compromised. In our experiments, the matrix (HCCA) melting point is 252°C [5]. The Ugi products are highly stable, due to their peptide-like amide bonds [6], and are reported to have melting points around 200°C [7, 8]. Simulations using OCHEM models [9, 10] predict the average melting and boiling points of the synthesized Ugi library to be around 113°C and 810°C, respectively.

- **Humidity:** Since both HCCA and the Ugi products are soluble and stable in water, individual spots should be able to take on water for a short period without affecting the data they store, though the samples should be dried prior to reading. The amide bonds in the Ugi products will slowly degrade by hydrolysis over time [11, 12]. This process can be drastically accelerated in the presence of certain enzymes [6]. As such, keeping the samples in a dry and sterile environment is recommended.

- **Light and radiation:** The Ugi products, specifically their amide bonds, are to some extent susceptible to dissociation under UV and X-ray radiation [13], and hence should be stored in a dark or LED lit room, without direct sunlight.

While a formal study of the storage lifetime is beyond the scope of this work, accelerated environmental testing of crystallized samples [14] could be performed at or below 100°C, with around 50% relative humidity, and under mild UV illumination to extrapolate dataset lifetimes.

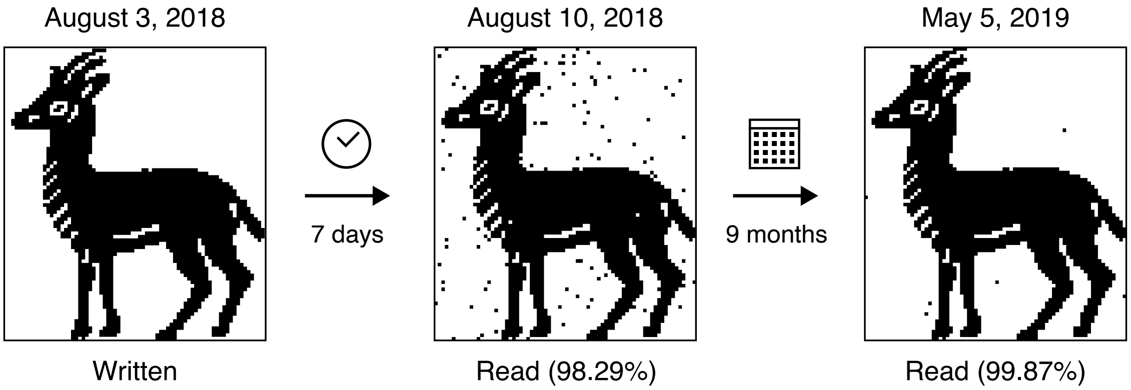

Supplementary Figure 8: Recovery of data after long-term storage. The binary image stored here was adapted from a 13th century Egyptian block print of an ibex [15]. In between measurements, the plate was stored in a transparent, unsealed cabinet in our mass spectrometry room at around 20°C. Note the lower accuracy of the earlier read was due to calibration issues with our mass spectrometer that were later resolved.

2.4. Comparison to Related Work

In this work, we store the largest amount of data among small-molecule information demonstrations to date. Even compared to more mature DNA memory, this work falls near the median of recent data storage examples. Figure 9 plots the total data encoded in over a dozen relevant demonstrations from the past ten years.

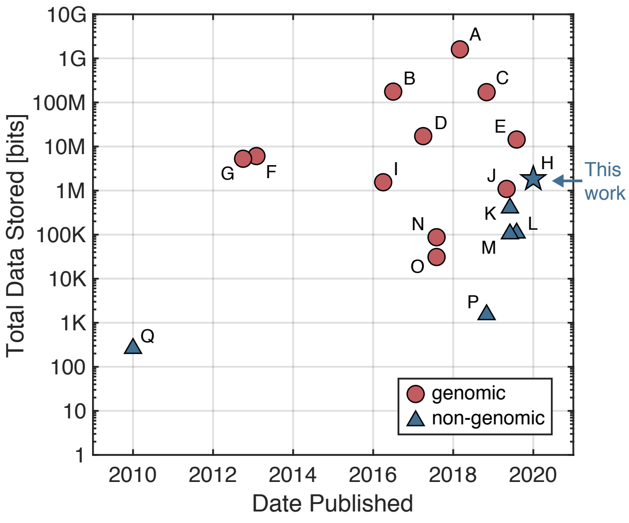

ID	1st Author	Published	Total [bits]	Chemistry
A	Organick	2018 (Feb.)	1,601,866,400	DNA
B	Blawat	2016 (Jun.)	176,000,000	DNA
C	Anavy	2018 (Oct.)	171,040,180	DNA
D	Erlich	2017 (Mar.)	17,174,528	DNA
E	Lopez	2019 (Jul.)	14,392,000	DNA
F	Goldman	2013 (Jan.)	6,056,408	DNA
G	Church	2012 (Sep.)	5,270,208	DNA
H	Arcadia	2019 (Dec.)*	1,844,527	Ugi products
I	Bornholt	2016 (Mar.)	1,544,000	DNA
J	Choi	2019 (Apr.)	1,090,032	DNA
K	Cafferty	2019 (May.)	402,880	peptides
L	Kennedy	2019 (Jul.)	108,692	metabolites
M	Rosenstein	2019 (May.)	104,111	Ugi products
N	Yazdi	2017 (Jul.)	87,152	DNA
O	Shipman	2017 (Jul.)	31,024	DNA
P	Martens	2018 (Oct.)	1,523	oligomers
Q	Ratner	2009 (Dec.)	261	aromatics

Supplementary Figure 9: Survey of recent chemical based storage demonstrations. **a** Total demonstrated storage over time, grouped by chemistry. **b** A table of molecular storage demonstrations [16–31]. *As a placeholder, this work is dated as being published at the end of 2019.

3. Mass Spectrometry

3.1. Spectrum Acquisition

To analyze the chemical makeup of many samples per day, we use a Fourier-transform ion cyclotron resonance (FT-ICR) mass spectrometer with matrix assisted laser desorption ionization (MALDI). During a measurement, a small fraction of a each spot's material is removed by a laser, and ions are excited into orbit in a high vacuum and a strong magnetic field. The orbital frequencies are a function of the mass and charge of each ion: $f = \frac{Bz}{2\pi m}$, where B is magnetic field strength [32]. The mass-to-charge ratio (m/z) of the ions can be found by taking the Fourier transform of the detected time-domain signal. Because the ions can be kept in orbit for several seconds, corresponding to millions of orbital cycles, FT-ICR mass spectra have exceptionally high resolution, often reaching peak widths of 0.001 Da or less. Here we typically acquire spectra for 1.5 seconds which results in a resolving power of 1.3×10^5 near 600 Da (Figure 15).

3.2. Mass Calibration

To ensure the mapping from cyclotron frequency to mass-to-charge ratio is done as accurately as possible, the mass spectrometer is calibrated with a sample having several known masses across the measurable range of 150-3000 Da. We perform this calibration prior to each MALDI plate measurement, using a solution of sodium trifluoroacetate (NaTFA): 0.05 $\mathrm{g\,L^{-1}}$ NaTFA dissolved in a 1:1 water:methanol mixture. The solution is injected into the instrument via electrospray ionization (ESI) and peaks are observed across the spectra which correspond to $\mathrm{Na_x(CF_3CO_2)_y}$. The measured positions of these peaks are then adjusted to their known values with a quadratic model, as are the rest of the m/z values. The results of an example m/z calibration are shown in Figure 10.

Additionally, a small offset calibration is performed within each spectrum using a common known mass. We spike the HCCA matrix solution with a manually purified Ugi product (F13) at a concentration of about 8 mM. This peak, commonly referred to as a mass lock, appears in all measured spectra, and is used to correct for fine offsets in each measured spectra, ± 0.01 Da or less.

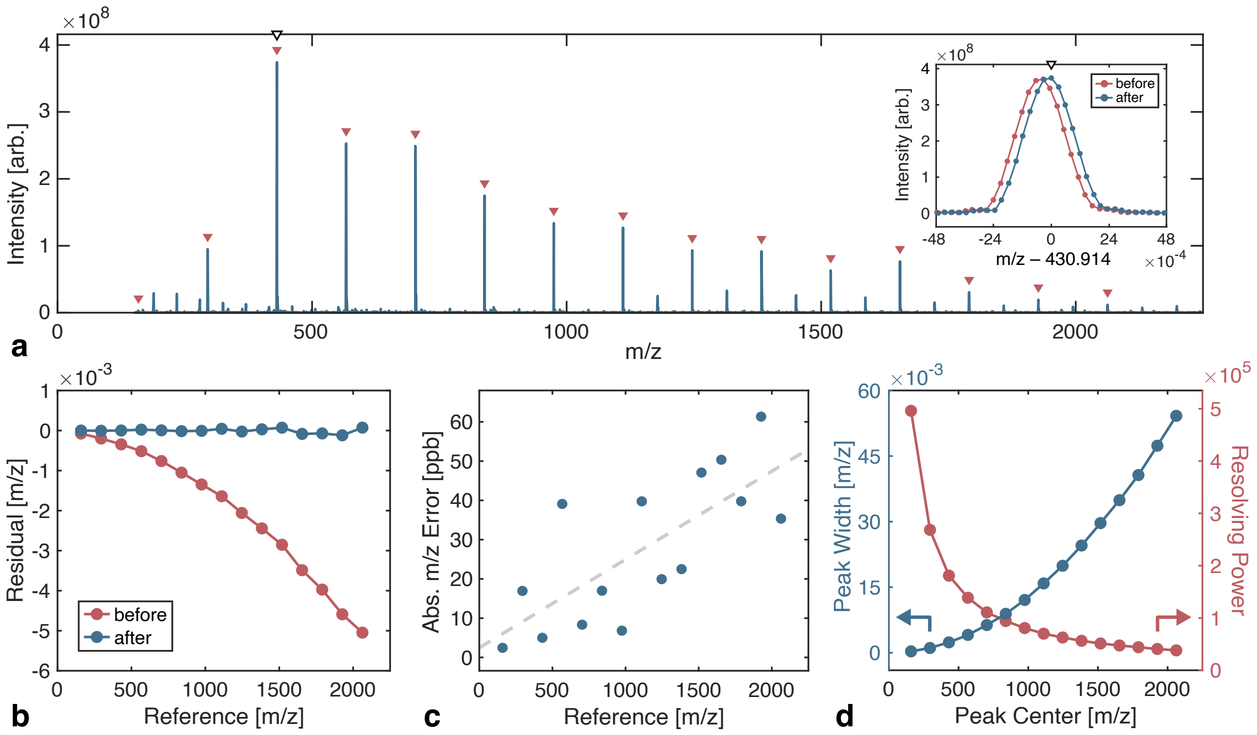

Supplementary Figure 10: A typical m/z calibration based on NaTFA and performed in electrospray ionization (ESI) mode. **a** The mass spectrum of NaTFA after calibration. The peaks used for calibration are marked with a triangle above them. The inset shows one of these peaks, both before and after calibration. **b** A plot of the m/z residuals, the difference between measured and expected, before and after quadratic calibration. **c** A plot of the absolute m/z error, in parts per billion (ppb): residual/reference $\times 10^9$, post-calibration. A linear trend-line, $y = 0.02248 \cdot x + 2.473$, is shown to emphasize the rise in error with increasing mass. **d** A plot, post-calibration, showing how peak width (FWHM) and resolving power (peak center over width) vary with m/z.

3.3. Spot Alignment

Instead of recording mass spectra over a perfectly rectangular grid, we minimize positioning errors in MALDI plate readout by imaging each plate with a flatbed scanner. We process the scanned images to automatically detect the precise location of spots using the Hough transform [33] (Figure 11). Standard spot addresses, such as “X01Y03”, are assigned by proximity to an expected grid. The resulting position map is loaded onto the mass spectrometer prior to running MALDI measurements.

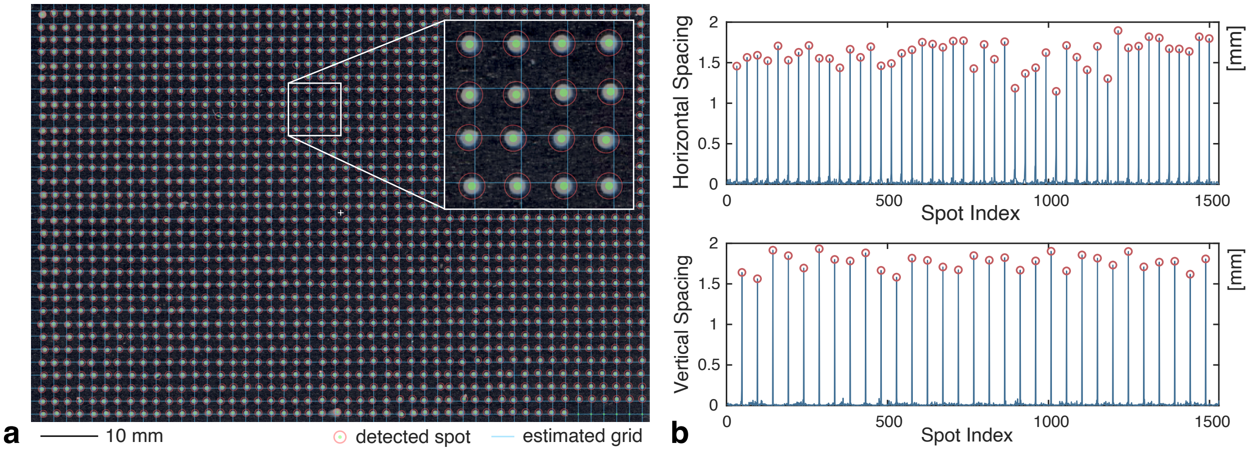

Supplementary Figure 11: MALDI Spot Alignment. **a** An image of a MALDI plate, containing the data mixtures for Anubis, highlighting the detected spots. The shown grid lines, averaged across each row and column, are used to assign each spot a standard plate address. **b** The horizontal (top) and vertical (bottom) spacing between the detected spots, as read from top-to-bottom and left-to-right. The start of rows and columns are marked with circles.

3.4. Repeated Reads

Each read from a MALDI spot removes a portion of the original sample. To quantify how a spot degrades after repeated measurement, we performed a series of spot burnout tests at various laser powers (Figure 12). The Ugi product (purified H6) and matrix concentrations in these samples were 1.56 mM and 88 mM, respectively. Each measurement was performed with 500 shots of the laser which was at medium focus and set to fire at 1000 Hz, while the scan region width was set to 500 μm. For most of the tested laser powers, the product could still be detected after 100 repeated reads.

Using the burned out region, we can estimate the amount of matrix material ionized per spectral recording. From the Anubis data plate, the height of a dried spot was coarsely measured, using in-plane microscope imaging, to be about 10 μm. Assuming the spots here are roughly the same height and that the majority of this was irradiated during the burn tests, then volume of the burned out region is 0.00239 mm^3. Since this volume was lost over the course of 100 reads, each measurement uses 23,900 μm^3 of the sample, which amounts to 0.24% of the total spot volume. Based on this ablation rate, the amount of Ugi product and matrix ionized per read is about 10 fmol (6 pg) and 13 pmol (2 ng), respectively. This approach could readily scale down to spatial dimensions of tens of microns without a loss in spectrum quality.

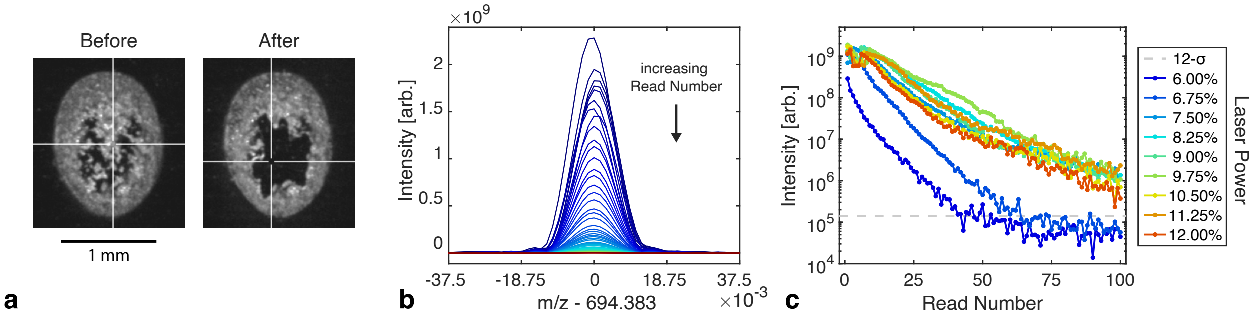

Supplementary Figure 12: A series of MALDI spot burnout tests performed at various laser powers. **a** Images of one of the spots before and after all 100 repeat reads at 12% power. The final ablated area, the dark region within the bright elliptical spot, is 0.239 mm^2 which is 24% of the total spot area. **b** The gradually weakening Ugi product peak (sodiated) as its spectrum is repeatedly measured at 9% laser power. **c** Ugi product peak intensity over the course of 100 reads. These repeated read tests were performed at differing laser powers, each with their own separate MALDI spot of identical composition. The dashed gray line, at 12-σ, represents a conservative bound for distinguishing signal peaks from background noise.

3.5. Signal-to-Noise Estimation

Spectral offset (μ) and background noise level (σ) are estimated as the mean and standard deviation, respectively, of the spectrum points below a certain intensity threshold. The signal cutoff is chosen to exclude strong peaks, which are effectively outliers, from the rest of a spectrum's points. We define this threshold (I_t), per spectra, as the upper Tukey fence for extreme outliers [34]:

$$I_T = Q_3 + 3 \cdot (Q_3 - Q_1) \tag{1}$$

where Q_1 and Q_3 are the first and third quartiles of spectrum intensity, respectively. The signal-to-noise ratio (SNR) is defined for each spectrum intensity I, as:

$$\mathrm{SNR}(I) = \frac{I - \mu}{\sigma} \tag{2}$$

where μ is the mean and σ is the standard deviation of background intensities, which are defined as those below the threshold: $I < I_T$. Using SNR instead of raw intensity tends to help normalize spectral recordings across measurement runs and plates, especially when different acquisition settings or sample concentrations are used. We generally find that peaks with SNR values at or above 12-σ can be confidently identified against the background.

3.6. Adduct Ions

Measured spectra frequently contain the sodiated (M+Na), protonated (M+H), and potassiated (M+K) variants of our Ugi products (see Figure 3 of the main text), but these make up a small fraction of the overall list of peaks in a MALDI mass spectrum. In general, it is difficult to predict the composition of adduct ions that will arise in a mixed sample. However, we can work backwards to try to identify some of the statistically useful features found with our supervised learning approaches.

To do so, we assembled a list of potential ions that may appear in our spectra based on adducts commonly seen in soft ionization [35], as well as those we have observed or suspect may form, such as adducts containing multiple molecular ions (nM), protons (H), common alkali metal ions (Na/K), matrix molecules (nHCCA), solvent (nDMSO), leftover reagents (Ami$X$/Ald$X$/Car$X$/Iso$X$), and other library compounds (nUgi$X$). In Figure 13 we plot the m/z features found with random forest regression, for three Ugi library elements, and tentatively label some of their prominent features. For example, the M+Na peak is useful for identifying the presence of Ugi 188, while Ugi 1439 is better identified by ion complexes containing unreacted starting materials. In all three plots, we find features that seem to correspond to other library elements, which we suspect is due to the competitive ionization of compounds in our mixed samples.

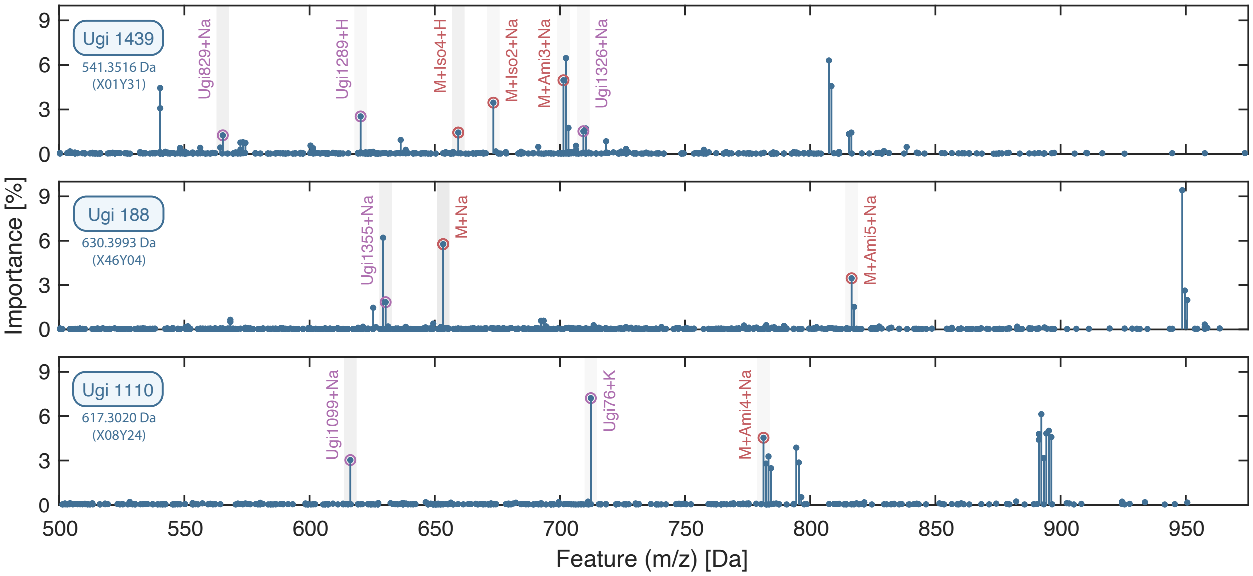

Supplementary Figure 13: Feature importance plots for three of the Ugi products used to encode data in the Violin experiment. Tentative assignments of ion adducts are highlighted, and were made using a mass tolerance of 0.005 Da and an importance cutoff of 1%. The PubChem IDs of the labeled starting reagents are: 75452 (Ami3), 66036 (Ami4), 2735655 (Ami5), 79129 (Iso2), 82558 (Iso4).

4. Parametric Sweeps

There are numerous experimental parameters that can be adjusted in these MALDI mass spectrometry measurements, such as acquisition duration and mass range, laser settings (power, diameter, frequency, shot count), scan settings (walk type, width, grid increment), and spot composition (sample and matrix concentrations, masses, spot size). Some of the tests that were done to select key parameters are shown here. Fortunately, the Ugi products in our library tend to respond similarly, and the majority of these parameters can be kept constant once a satisfactory configuration is found. However, to account for run-to-run and plate-to-plate variability, and to ensure high signal-to-noise ratios, a few parameters, such as laser shot count and power, are often manually tuned before measuring an entire plate, by selecting a single spot on the plate and tuning its total ion current (TIC) to the $10^8 - 10^9$ range. If the TIC is made much higher than this, spectra begin to degrade due to increased ion-ion interactions [36].

4.1. Spot Size

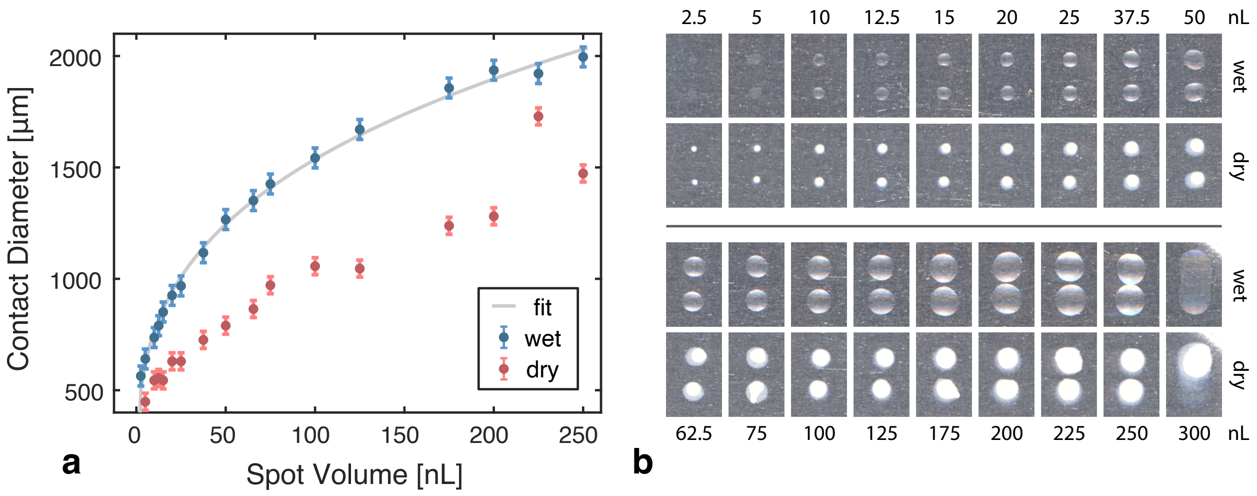

Supplementary Figure 14: Contact diameter as a function of droplet volume. **a** A graph of measured contact area, of the top-most spots in b, as a function of volume spotted. For the freshly spotted and still wet droplets, a fit to a pseudo-hemispherical model is shown: $d = (a \cdot \frac{12}{\pi} \cdot V)^{\frac{1}{3} \cdot b}$, where $a = 8.009 \times 10^7$ and $b = 0.9117$. The diameters of the dried spots are also shown. **b** Scanned images of the spots, both after spotting and after drying, for each volume tested. The spot sizes were measured from these images using ImageJ [37]. The dimensions of the shown rectangular windows are 3.756 x 5.673 mm. The spots were made of 176.2 mM HCCA in DMSO and were placed on a 1536-well grid (2.25 mm pitch).

4.2. Acquisition Duration

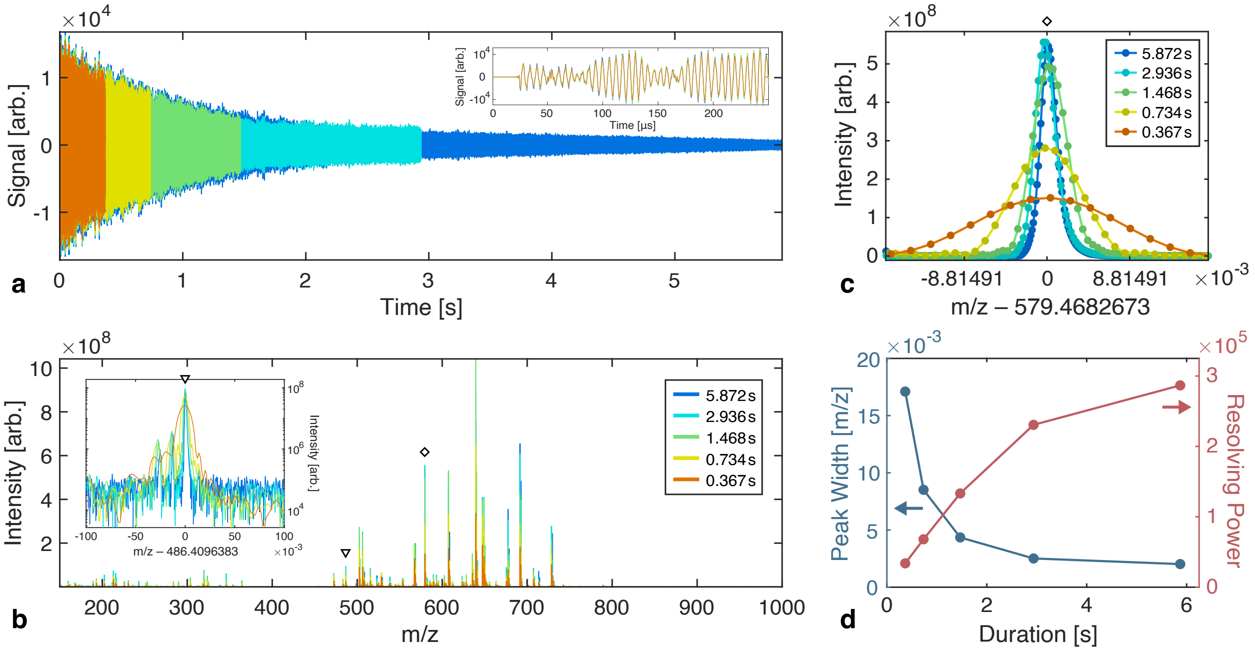

Supplementary Figure 15: Varying FT-ICR mass spectrum acquisition time for a single MALDI sample. **a** Raw time-domain signals acquired at various durations (see the legend in c or b). The inset shows the first 250 μs of the signals, which overlap due to phase locking. **b** The spectra resulting from transforming the above raw signals into the mass-to-charge domain. An inset with intensity on a log-scale is shown to highlight the widening of peaks with decreasing measurement time. **c** A narrow region from the spectra shown in b, which depicts how runtime affects the shape of a compound's peak. **d** Peak width (FWHM) and resolving power for each acquisition duration, as measured from the traces shown in c.

4.3. Laser Power

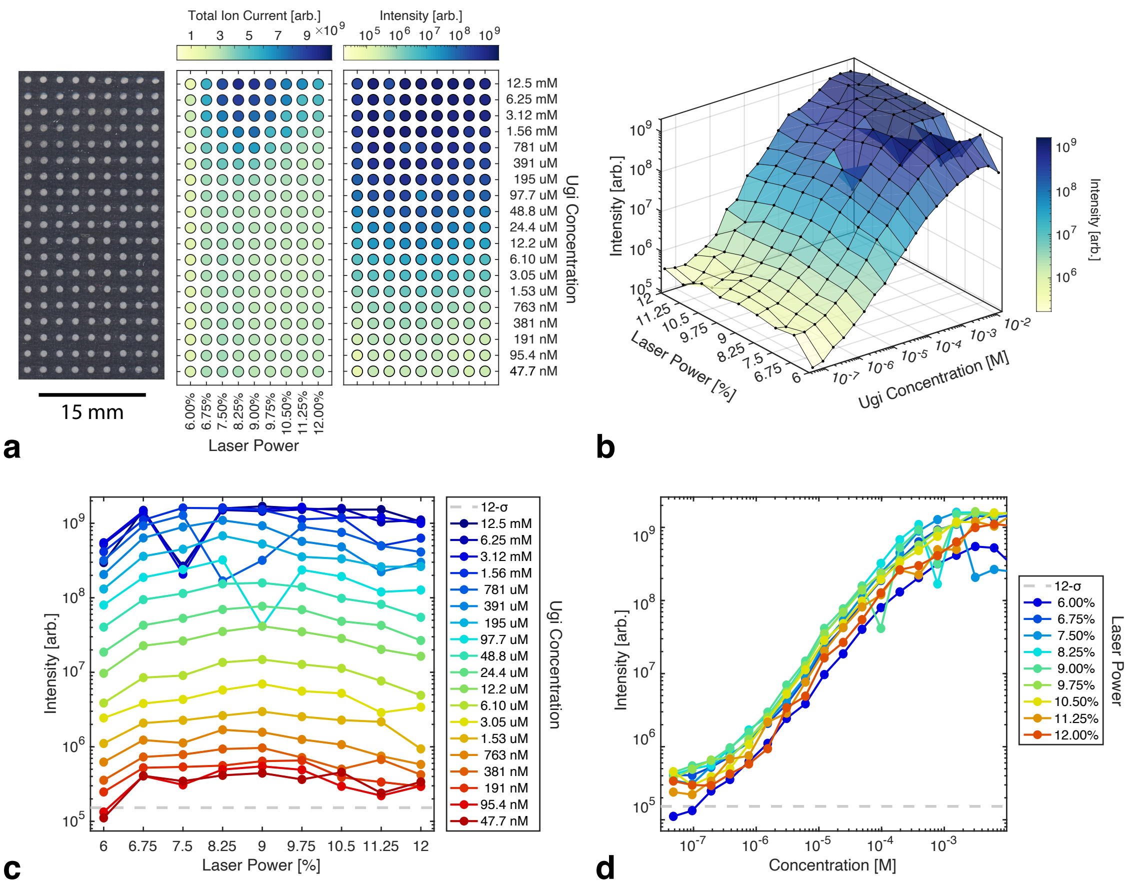

Supplementary Figure 16: A study sweeping laser power and Ugi product (H6) concentration. **a** A collage of the tested samples showing, from left to right, an image of the crystallized MALDI spots, a total ion current (TIC) map for each spot's spectra, and an intensity map for the sodiated peak of the swept Ugi product. Each tested laser power and Ugi product concentration can be read from the given axis. **b** A 3-D surface visualization of the Ugi product's sodiated peak intensity across the swept parameter space. **c** A plot of peak intensity as a function of laser power for each tested Ugi concentration. **d** A plot of peak intensity as a function of Ugi concentration for each tested laser power.

4.4. Ugi Product Concentration

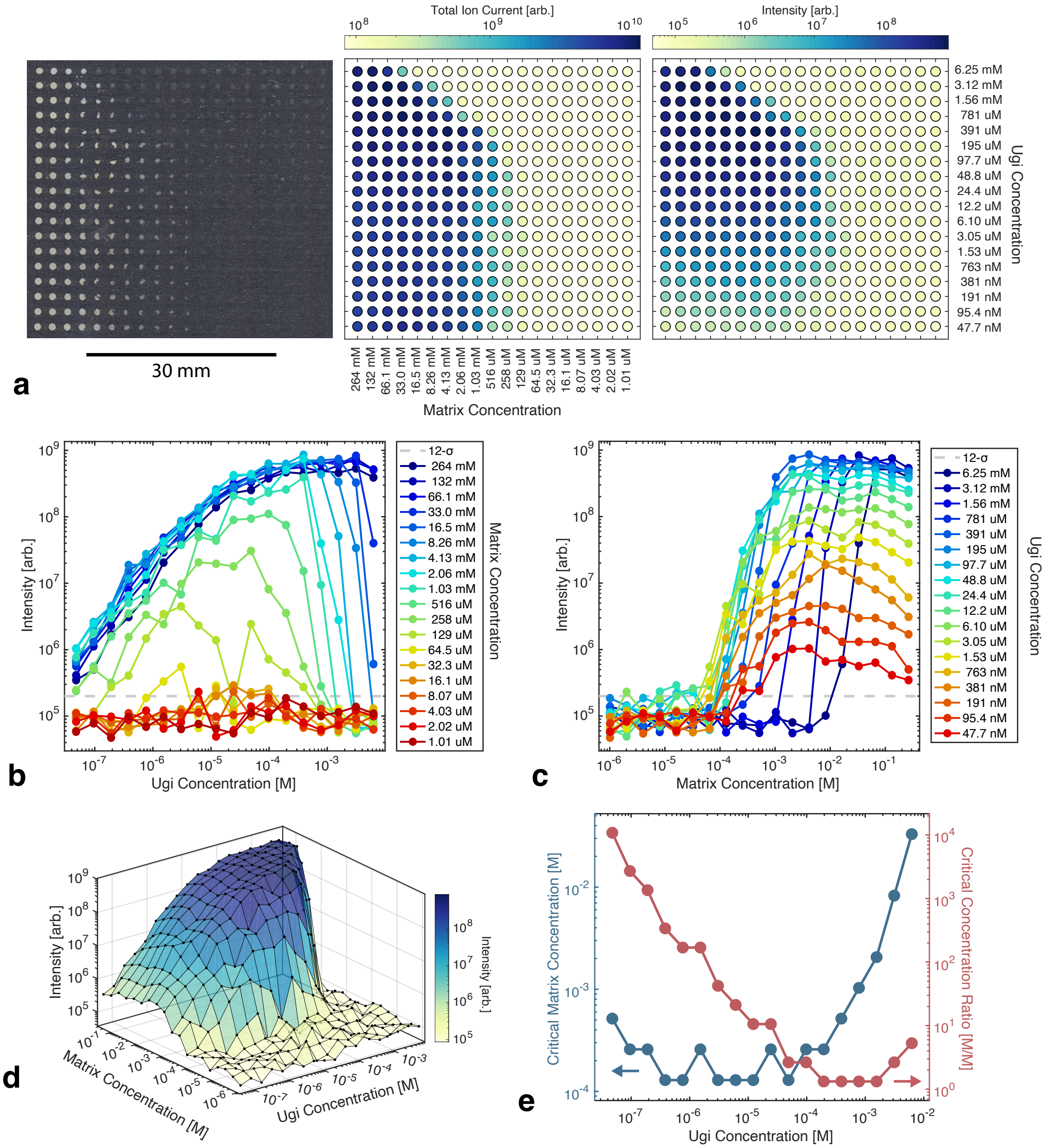

Supplementary Figure 17: A study sweeping matrix (HCCA) and Ugi product (H6) concentration. **a** A collage of the tested samples showing, from left to right, an image of the crystallized MALDI spots, a total ion current (TIC) map for each spot's spectra, and an intensity map for the sodiated peak of the swept Ugi product. Each tested matrix and Ugi product concentration can be read from the given axis. **b** A plot of peak intensity as a function of Ugi concentration for each tested matrix concentration. **c** A plot of peak intensity as a function of matrix concentration for each tested Ugi concentration. **d** A 3-D surface visualization of the sodiated Ugi product peak's intensity across the swept parameter space. **e** The critical matrix concentrations and ratios for each Ugi compound concentration. The critical values were found as the log-scale midpoint between the high and low intensity regimes of the traces shown in c.

Supplementary References

1. Unknown artist. *Book of the Dead for the Singer of Amun, Nany.* Accession 30.3.31. Metropolitan Museum of Art, NY, USA. (ca. 1050 B.C.)
2. Unknown artist. *Leopard Bearing Lion's Order to Fellow Judges.* Accession 1981.373.51. Metropolitan Museum of Art, NY, USA. (18th century).
3. Baldung, H. *Angels Served at a Table.* Accession 17.3.3034. Metropolitan Museum of Art, NY, USA. (1507).
4. Picasso, P. *Violin.* © Estate of Pablo Picasso / Artists Rights Society (ARS), New York.
5. Tarzi, O. I., Nonami, H. & Erra-Balsells, R. The effect of temperature on the stability of compounds used as UV-MALDI-MS matrix: 2, 5-dihydroxybenzoic acid, 2, 4, 6-trihydroxyacetophenone, α-cyano-4-hydroxycinnamic acid, 3, 5-dimethoxy-4-hydroxycinnamic acid, nor-harmane and harmane. *Journal of mass spectrometry* **44,** 260–277 (2009).
6. Mahesh, S., Tang, K.-C. & Raj, M. Amide Bond Activation of Biological Molecules. *Molecules* **23,** 2615 (2018).
7. Otaibi, A. A. *et al.* A methanol and protic ionic liquid Ugi multicomponent reaction path to cytotoxic α-phenylacetamido amides. *RSC Advances* **9,** 7652–7663 (2019).
8. Rezayan, A. H. *et al.* Synthesis of Novel Fluorene Bisamide Derivatives via Ugi Reaction and Evaluation their Biological Activity against Mycobacterium species. *Iranian journal of pharmaceutical research : IJPR* **16,** 745–755 (2017).
9. Tetko, I. V., Lowe, D. M. & Williams, A. J. The development of models to predict melting and pyrolysis point data associated with several hundred thousand compounds mined from PATENTS. *Journal of Cheminformatics* **8,** 2 (2016).
10. Brandmaier, S., Sahlin, U., Tetko, I. V. & Öberg, T. PLS-Optimal: A Stepwise D-Optimal Design Based on Latent Variables. *Journal of Chemical Information and Modeling* **52,** 975–983 (2012).
11. Snape, T. J., Astles, A. M. & Davies, J. Understanding the chemical basis of drug stability and degradation. *Pharmaceutical Journal* **285,** 416–417 (2010).
12. Radzicka, A. & Wolfenden, R. Rates of Uncatalyzed Peptide Bond Hydrolysis in Neutral Solution and the Transition State Affinities of Proteases. *Journal of the American Chemical Society* **118,** 6105–6109 (1996).
13. Johnson, P. S. *et al.* Universal mechanism for breaking amide bonds by ionizing radiation. *The Journal of Chemical Physics* **135,** 044702 (2011).
14. Escobar, L. A. & Meeker, W. Q. A Review of Accelerated Test Models. *Statistical Science* **21,** 552–577 (2007).
15. Unknown artist. *Ibex or Gazelle, Block Print.* Accession 2016.624. The Metropolitan Museum of Art, NY, USA. (13th or 14th century).
16. Organick, L. *et al.* Random access in large-scale DNA data storage. *Nature biotechnology* **36,** 242 (2018).

17. Blawat, M. *et al.* Forward Error Correction for DNA Data Storage. *Procedia Computer Science* **80,** 1011–1022 (2016).

18. Anavy, L., Vaknin, I., Atar, O., Amit, R. & Yakhini, Z. Improved DNA based storage capacity and fidelity using composite DNA letters. Preprint at `https://www.biorxiv.org/content/10.1101/433524v1` (2018).

19. Erlich, Y. & Zielinski, D. DNA Fountain enables a robust and efficient storage architecture. *Science* **355,** 950–954 (2017).

20. Lopez, R. *et al.* DNA assembly for nanopore data storage readout. *Nature Communications* **10,** 2933 (2019).

21. Goldman, N. *et al.* Towards practical, high-capacity, low-maintenance information storage in synthesized DNA. *Nature* **494,** 77 (2013).

22. Church, G. M., Gao, Y. & Kosuri, S. Next-generation digital information storage in DNA. *Science* **337,** 1628–1628 (2012).

23. Bornholt, J. *et al.* A DNA-Based Archival Storage System. *ACM SIGOPS Operating Systems Review* **50,** 637–649 (2016).

24. Choi, Y. *et al.* High information capacity DNA-based data storage with augmented encoding characters using degenerate bases. *Scientific Reports* **9,** 6582 (2019).

25. Kennedy Eamonn andArcadia, C. E. *et al.* Encoding information in synthetic metabolomes. *PLOS ONE* **14,** 1–12 (2019).

26. Rosenstein, J. K. *et al.* Principles of Information Storage in Small-Molecule Mixtures. Preprint at `https://arxiv.org/abs/1905.02187` (2019).

27. Yazdi, S. M. H. T., Gabrys, R. & Milenkovic, O. Portable and Error-Free DNA-Based Data Storage. *Scientific Reports* **7,** 5011 (2017).

28. Cafferty, B. J. *et al.* Storage of Information Using Small Organic Molecules. *ACS Central Science* (2019).

29. Shipman, S. L., Nivala, J., Macklis, J. D. & Church, G. M. CRISPR–Cas encoding of a digital movie into the genomes of a population of living bacteria. *Nature* **547,** 345 (2017).

30. Martens, S. *et al.* Multifunctional sequence-defined macromolecules for chemical data storage. *Nature communications* **9,** 4451 (2018).

31. Ratner, T., Reany, O. & Keinan, E. Encoding and processing of alphanumeric information by chemical mixtures. *ChemPhysChem* **10,** 3303–3309 (2009).

32. Amster, I. J. Fourier Transform Mass Spectrometry. *Journal of Mass Spectrometry* **31,** 1325–1337 (1996).

33. Atherton, T. J. & Kerbyson, D. J. Size invariant circle detection. *Image and Vision Computing* **17,** 795–803 (1999).

34. Hoaglin, D. C., Iglewicz, B. & Tukey, J. W. Performance of Some Resistant Rules for Outlier Labeling. *Journal of the American Statistical Association* **81,** 991–999 (1986).

35. Huang, N., Siegel, M. M., Kruppa, G. H. & Laukien, F. H. Automation of a Fourier transform ion cyclotron resonance mass spectrometer for acquisition, analysis, and e-mailing of high-resolution exact-mass electrospray ionization mass spectral data. *Journal of the American Society for Mass Spectrometry* **10,** 1166–1173 (1999).
36. Masselon, C., Tolmachev, A. V., Anderson, G. A., Harkewicz, R. & Smith, R. D. Mass measurement errors caused by "local" frequency perturbations in FTICR mass spectrometry. *Journal of the American Society for Mass Spectrometry* **13,** 99–106 (2002).
37. Schneider, C. A., Rasband, W. S. & Eliceiri, K. W. NIH Image to ImageJ: 25 years of image analysis. *Nature Methods* **9,** 671 (2012).
